# Supplementary material for: The role of hydration in the removal of glyphosate (GLY) and aminomethylphosphonic acid (AMPA) by nanofiltration membranes
Source: Nat Commun. 2026 Apr 23;17:3741. doi: 10.1038/s41467-026-71492-y (PMC13106632; doi:10.1038/s41467-026-71492-y)
Supplement: Supplementary file 1 — Supplementary Information [file 41467_2026_71492_MOESM1_ESM.pdf]

SUPPORTING INFORMATION

The role of hydration in the removal of glyphosate (GLY) and aminomethylphosphonic acid (AMPA) by nanofiltration membranes

Phuong B. Trinh[1], Minh N. Nguyen[1], Zdenek Futera[2], Babak Minofar[3], Marco Personeni[4], Poul B. Petersen[4], Andrea I. Schäfer[1]*

[1]Institute for Advanced Membrane Technology (IAMT), Karlsruhe Institute of Technology (KIT), Hermann-von-Helmholtz-Platz 1, 76344 Eggenstein-Leopoldshafen, Germany

[2] Department of Physics, Faculty of Science, University of South Bohemia in České Budějovice, Branišovská 1760, 370 05 České Budějovice, Czech Republic

[3]Department of Physical Chemistry, Faculty of Chemistry, University of Lodz, Tamka 12, 91-403 Lodz, Poland

[4]Physikalische Chemie II, Ruhr-Universität Bochum, Universitätsstraße 150, 44801 Bochum, Germany

*Corresponding author: Andrea.Iris.Schaefer@kit.edu (A. I. Schäfer), +49 (0)721 608 26906

Supplementary Discussion 1. Donnan exclusion of GLY/AMPA in NF

Charge (Donnan) exclusion indicates the retention due to charge interactions (electrostatic repulsion) between the charged ions or micropollutants and the charged surface of the membrane [1]. The charge at the membrane surface is characterized by the zeta potential ζ [2] and the double layer thickness of charge solutes on membrane surface (Debye length, κ^{-1}) [3, 4] (Supplementary Figure 1).

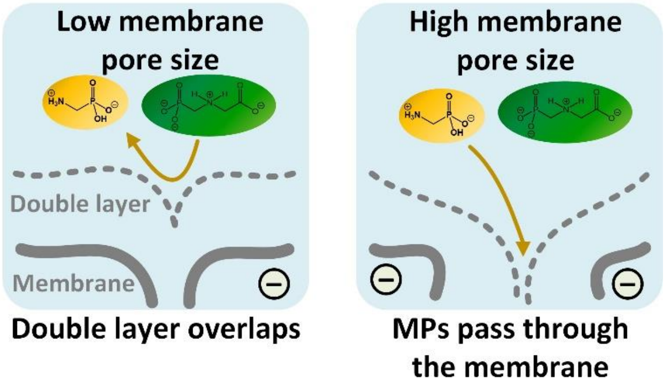

Supplementary Figure 1. Double layer of membrane with small and large pore size in the same ionic strength solution.

The relativeness between the pore radius and the Debye length controls the transport of ions and charged species that are smaller than membrane pores [3, 4]. If the Debye length is greater than the pore radius, all charged species can interact with the charged pore wall; this does not mean they are all retained, but these charged species encounter electrostatic resistance that depends on the (pore) surface charge [5]. If Debye length is smaller than the pore radius, species in the pore center will pass through the pores without electrostatic interaction with the pore wall.

Supplementary Discussion 2. Speciation of GLY and AMPA

GLY and AMPA are highly dependent on pH due to the amine, phosphate, and carboxyl [6, 7]. The speciation of GLY and AMPA at different pH was shown in Supplementary Figure 2.

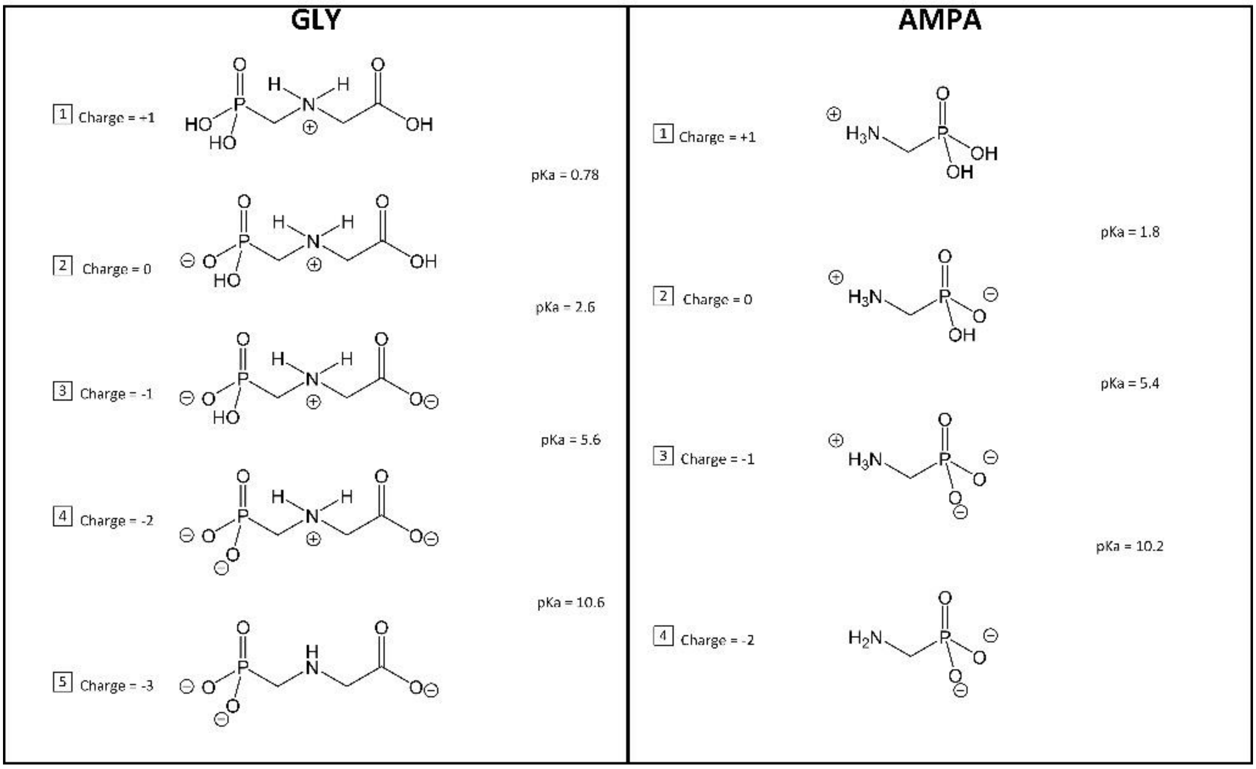

Supplementary Figure 2. Speciation of GLY and AMPA at pH 2 – 12 (calculated by Visual MINTEQ (v 3.1, KTH, Sweden) at the corresponding water matrix).

At pH 7-8 (environmentally relevant), the charge of GLY is –2 while the charge of AMPA is –1. With pH varying from 2 to 12, the charge of GLY varies from 0 to –3 and the charge of AMPA from 0 to –2. Even with zero charge, GLY and AMPA are in zwitterionic forms at pH 2 and pH 4, which means charge interactions are relevant at all pH values [8].

Supplementary Discussion 3. GLY and AMPA removal by BW 30 at different pH

The dense membrane BW 30 (MWCO 100 Da) was evaluated for GLY and AMPA removal at different pH values (Supplementary Figure 3).

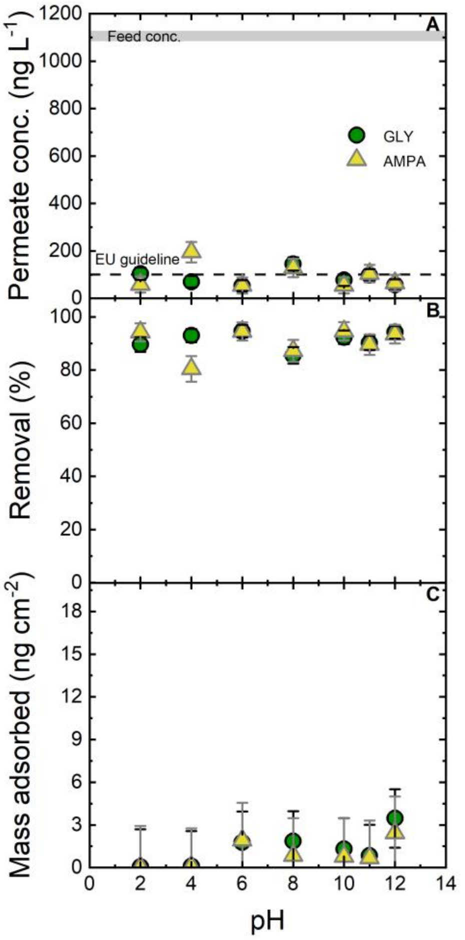

Supplementary Figure 3. Permeate concentration (A), removal (B) and mass loss (C) as a function of pH with GLY/AMPA removal by BW 30 membrane (BW 30 membrane, flux 30 L m^{-2} h^{-1}, initial GLY/AMPA concentration 1 µg L^{-1}, 1mM $NaHCO_3$, 10 mM NaCl, 20 ^{o}C).

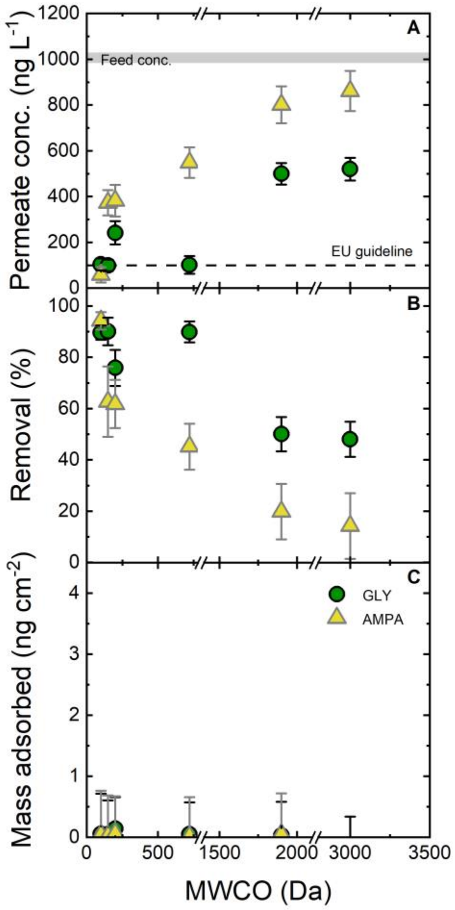

Supplementary Figure 4. Permeate concentration (A), removal (B) and mass loss (C) as a function of membrane MWCO (flux 50 L m^{-2} h^{-1}, initial GLY/AMPA concentration 1 µg L^{-1}, 1mM $NaHCO_3$, 10 mM NaCl, 20 ^{o}C, pH 2 ± 0.1). Flux for BW 30 and HY 70 was 30 L m^{-2} h^{-1}

At flux 30 L m^{-2} h^{-1}, no significant change in GLY and AMPA removal was observed, and in the range of 90% for both herbicides. The full retention of GLY and AMPA was obtained by BW 30 membrane.

Supplementary Discussion 4. GLY/AMPA removal by different membranes at pH 2

To evaluate the filtration of GLY and AMPA at pH 2, 6 membranes (BW 30, NF 90, NF 270, HY 70, HY 50, HY 10) with the MWCO in the range of 100–3000 Da were chosen (Supplementary Figure 4).

Removal of GLY and AMPA with the increase of MWCO. Removal of GLY decreased from 90% to 48% while increasing MWCO from 100 to 3000 Da. Removal of AMPA decreased from 90% to 14% at the loose membrane.

Supplementary Discussion 5. GLY and AMPA removal by loose membrane at different pH

The loose membrane HY 70 (MWCO 720 Da) and HY 10 (MWCO 3000 Da) were evaluated for GLY and AMPA removal (Supplementary Figure 5). The surface charge of HY 70 and HY 10 becomes more negative at higher pH [9].

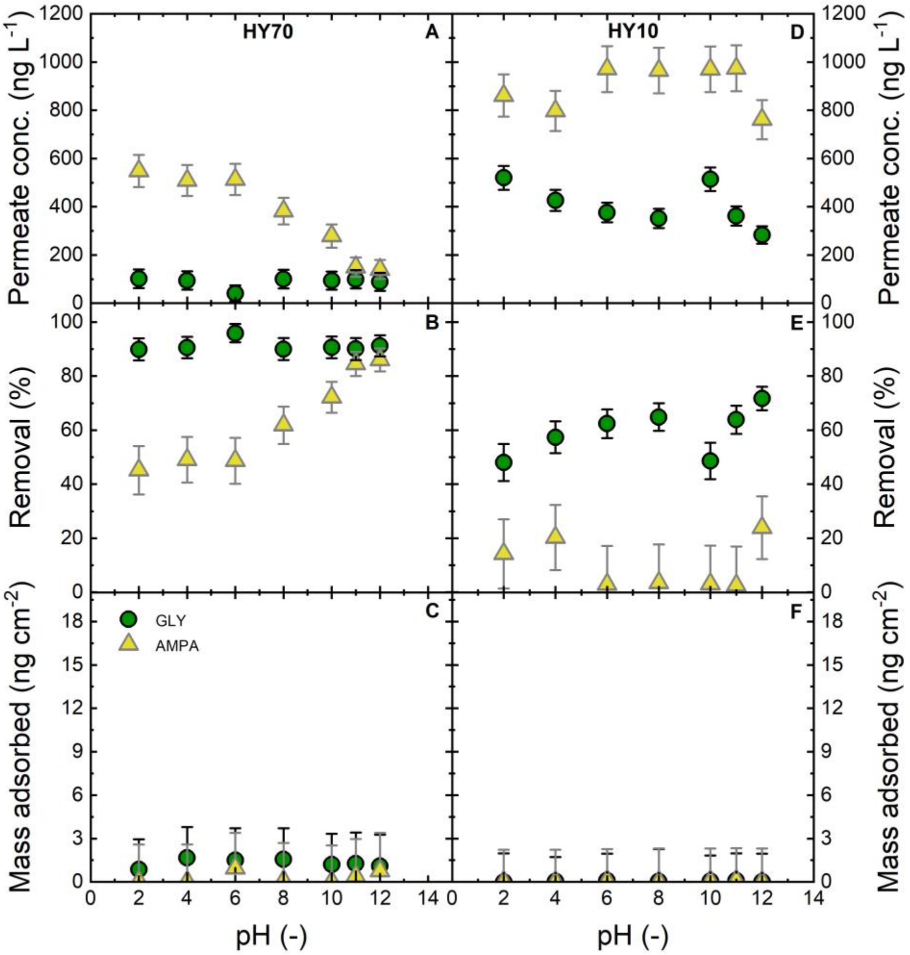

Supplementary Figure 5. Permeate concentration (A), removal (B) and mass loss (C) as a function of pH with GLY/AMPA removal by HY 70 and HY 10 membrane (HY 70 flux 30 L m^{-2} h^{-1}, HY 10 flux 50 L m^{-2} h^{-1}, initial GLY/AMPA concentration 1 µg L^{-1}, 1mM $NaHCO_3$, 10 mM NaCl, 20 ^{o}C).

GLY removal increased with the increase of pH for the HY 10 membrane. GLY removal increased from 48% to 71% by HY 10. AMPA removal was low and remained at 11–14% at all pH with HY 10 membrane (loosest membrane MWCO 3000 Da). For HY 70, GLY removal stayed constant at 90% for all pH (might be due to lower flux 30 L m^{-2} h^{-1}) while AMPA removal was constant at pH 2–6 then increased from 45% to 85% when pH increased from 2 to 12.

Supplementary Discussion 6. Concentration polarization in nanofiltration membranes

The rejection of GLY and AMPA is also controlled by concentration polarization [7]. Therefore, the concentration polarization is evaluated via solute flux, concentration at the membrane surface, and real removal of the NF membranes.

The GLY/ AMPA external mass transfer, which is characterized by the external mass transfer coefficient k_m, controls the transport of GLY and AMPA from the bulk solution to the membrane surface. It plays an important role in the concentration polarization and rejection of solute by NF membranes [10]. The external mass transfer coefficient k_m can be calculated from Sherwood number as represented in (S1)). [11, 12].

$$Sh = \frac{k_m \cdot D_h}{D} = a \cdot Re^b \cdot Sc^c \qquad \text{(S1)}$$

where D_h is the hydraulic diameter of the stirred cell (m), D is the diffusion coefficient (m^2 s^{-1}), Re and Sc are the dimensionless Reynolds and Schmidt numbers ($Sc = \nu/D$ where ν (m^2 s^{-1}) is the kinematic viscosity), respectively; a, b, c are adjustable dimensionless parameters, which are dependent on system geometry and laminar or turbulent conditions [13].

For the stirred cell system in this study, Sh and Re are calculated using the following equation [14, 15].

If Re < 30000 (laminar)

$$Sh = 0.285 \cdot Re^{0.567} \cdot Sc^{0.33} \qquad \text{(S2)}$$

If 32000 < Re > 82000 (turbulent)

$$Sh = 0.044 \cdot Re^{0.75} \cdot Sc^{0.33} \qquad \text{(S3)}$$

For stirred cells

$$Re = \frac{\omega \cdot r^2}{\nu} \qquad \text{(S4)}$$

where ω (rad/s) is the agitator speed and $r = \frac{1}{2} D_h$ is the radius of the stirred cell base. The concentration at the membrane surface is calculated as follows

$$c_m = c_b \left((1 - R_{obs}) + R_{obs} \cdot e^{\frac{J_v}{k_m}} \right) \qquad \text{(S5)}$$

where c_b is the concentration at the bulk solution (ng L^{-1}), R_{obs} is the observed retention [10]. The solute flux J_S is calculated as

$$J_S = J_v \cdot c_p \qquad \text{(S6)}$$

where c_p (ng L^{-1}) is the permeate concentration and J_v is the water flux (m s^{-1}).

Supplementary Discussion 7. Compression of hydration layer in membrane pores

To estimate the compression of hydration in membrane pores, the solvated species are considered as spherical particles of approximate radius 0.5 nm. In the bulk solution, the hydration layer is complete, as can be seen in Supplementary Figure 6.

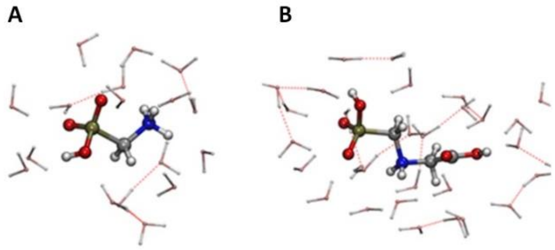

Supplementary Figure 6. Hydration shell of charge-neutral (a) AMPA, and (b) GLY in the bulk water solution (snapshot from MD simulations, hydrogen bonds are indicated by the dashed red lines)

Application of 70 bar pressure would shrink the hydrogen bonds between the species and the water molecules by ~0.01 Å, using the typical hydrogen-bond force constant value of 1 eV A^{-2} (or 96 kJ mol^{-1} per A^2). Indeed, this compression is negligible in the bulk solution.

However, when the species enters the membrane pores, it interacts with polar groups that naturally replace some of the hydration-shell water molecules. This is what dehydration means: some water molecules move away from the species' proximity shell and towards the 'bulk' (*i.e.* far away from the species). This process is spontaneous, and the species tends to stick to the pore surface and form clusters as they move through it. That is shown in the following snapshots from the molecular dynamics simulations (Supplementary Figure 7).

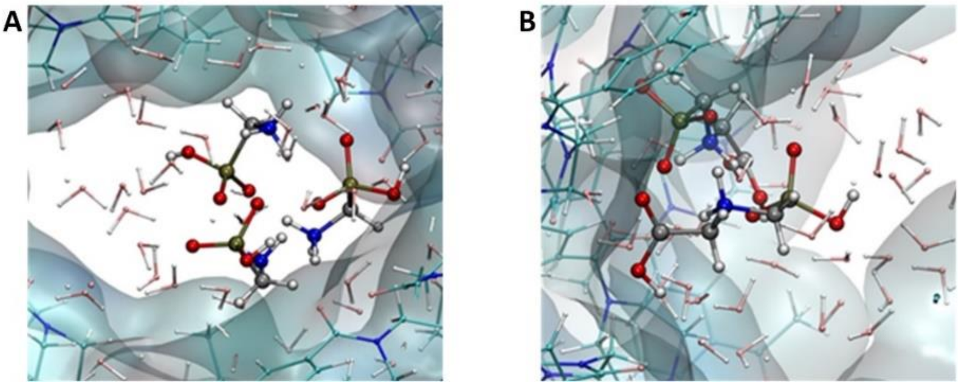

Supplementary Figure 7. Hydration shell of charge-neutral (a) AMPA, and (b) GLY inside the piperazine-polyamide membrane pores (snapshot from molecular dynamics simulations, pore surface around the polyamide is drawn)

In this regard, the transport of ion/species does not depend on the compression of the hydration shell, but the reordering or shuffling of the hydrogen bonding network. In the bulk phase, the energy of this rearrangement is similar to the hydrogen bond strength and in the order of 20 kJ mol^{-1} [16]. In the membrane system, this energy is higher but within the same order.

Epsztein *et al.* summarized that the 'dehydration energy' for ion permeation in NF 270 and NF 90 membranes derived from experiments is below 65 kJ mol^{-1} [17]. This energy can be explained as the required energy when some water molecules shred hydrogen bonds with each other in the hydration layer, subtracted by the released energy when they form hydrogen bonds in the bulk phase. The pressure is therefore not required for changing the volume of the system *i.e.* compression, but to contribute to the molecular kinetics (facilitating solution flow through the membrane pores and helping the species to get into contact with the pore surface) and speed up the hydrogen bond network reordering.

Supplementary Discussion 8. Hydration shell of GLY and AMPA

The RDFs were computed for water oxygen and hydrogen interactions with the carboxyl group of GLY and the amino group of AMPA (Supplementary Figure 8).

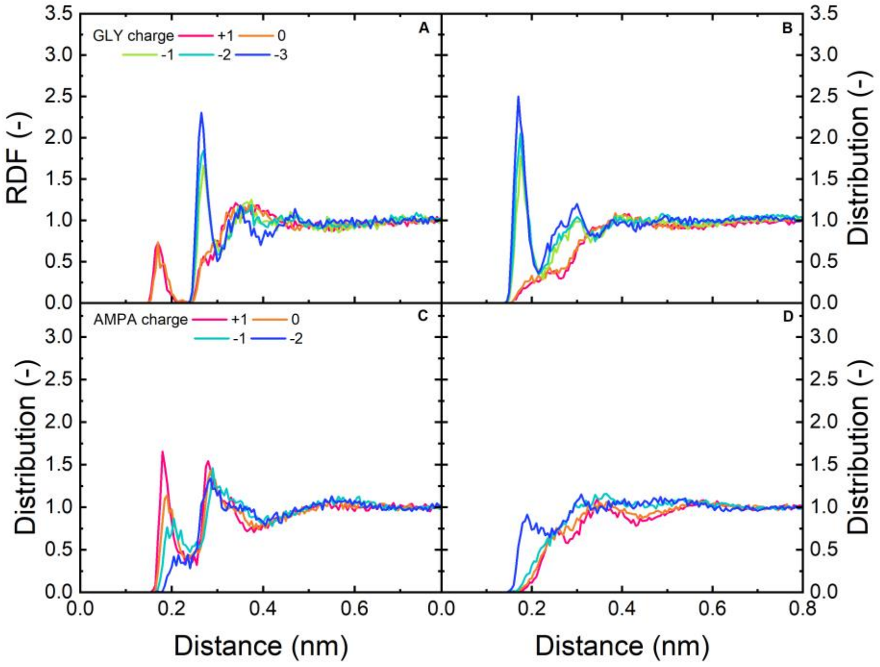

Supplementary Figure 8. Radial distribution functions (RDF) detect interactions between GLY carboxyl groups (A), AMPA amino groups (B) water oxygen (left) and hydrogen (right) atoms. The coordination numbers, corresponding to RDF integrals, are shown in lower panels.

The carboxyl group of GLY interacts with the water oxygen via its hydrogen atom at the +1 and 0 charges (corresponding to low pH < 4 when the carboxyl group is protonated). At higher pH, the carboxylate group is deprotonated, showing the charge –1 to –3, and H-bonding was formed between the carboxylate oxygens and water hydrogens. The water interaction with NH_3 is weaker than the phosphate group unless the group gets deprotonated in charge –2 (pH > 10), where the amine group interacts with nearby water hydrogen.

Supplementary Method 9. Experimental parameters

Filtration by BW 30 membrane at varying pH

Pressure, flux, temperature, and permeate concentration in filtration experiments were recorded with different feed pH.

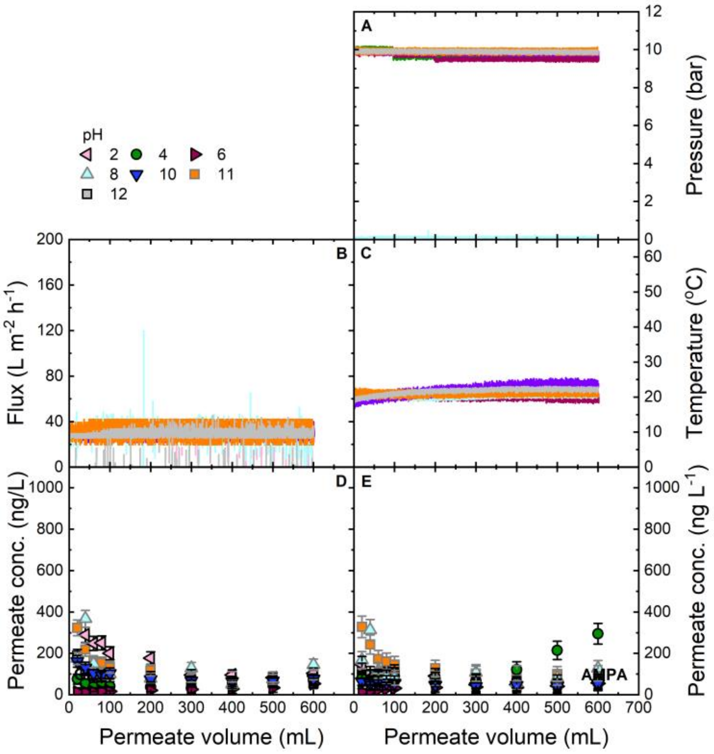

Supplementary Figure 9. Pressure, flux, feed temperature, permeate concentration by BW30 membrane (flux 30 L m^{-2} h^{-1}, initial GLY/AMPA concentration 1 µg L^{-1}, 1mM $NaHCO_3$, 10 mM NaCl, 20 ± 0.5 °C).

The pressure was stable over time. Temperature variation was within 0.5 °C in each experiment. The flux had a maximum variation of 10%. The permeate concentration decreased in the first 100 mL, then remained stable until the end of the filtration.

Filtration by NF 90 at varying pH

Pressure, flux, temperature, and permeate concentration in filtration experiments were recorded with different feed pH.

The pressure was stable over time. Temperature variation was within 0.5 °C in each experiment. The flux had a maximum variation of 10%. The permeate concentration decreased in the first 100 mL then remained stable until the end of the filtration.

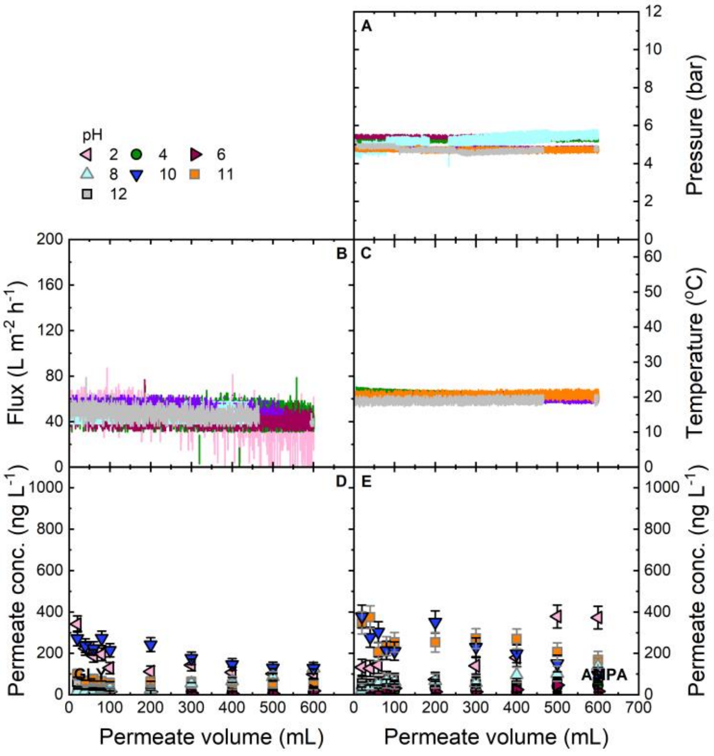

Supplementary Figure 10. Pressure, flux, feed temperature, permeate concentration by NF90 membrane (flux 50 L m^{-2} h^{-1}, initial GLY/AMPA concentration 1 μg L^{-1}, 1mM $NaHCO_3$, 10 mM NaCl, 20 ± 0.5 °C).

Filtration by NF 270 at varying pH

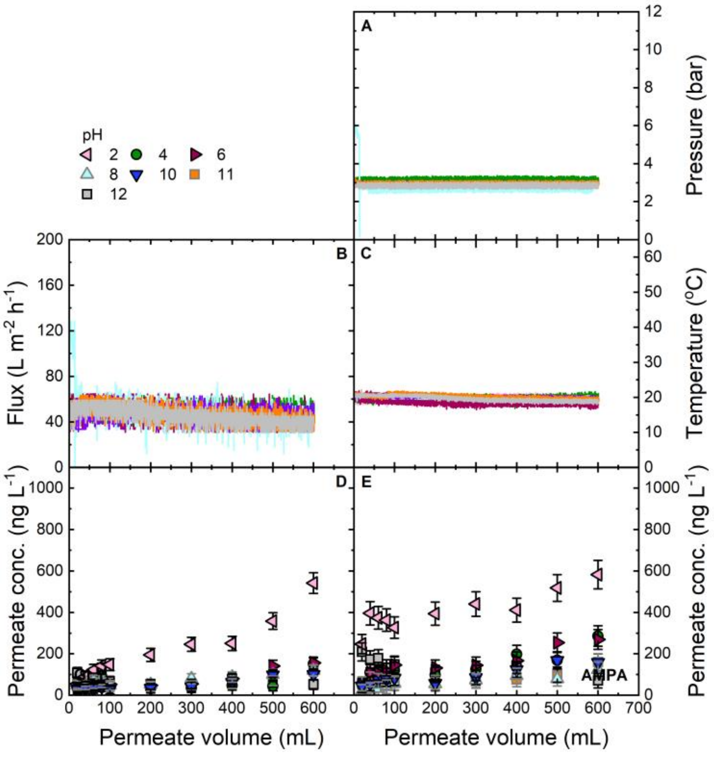

Supplementary Figure 11. Pressure, flux, feed temperature, permeate concentration by NF270 membrane (flux 50 L m^{-2} h^{-1}, initial GLY/AMPA concentration 1 μg L^{-1}, 1mM $NaHCO_3$, 10 mM NaCl, 20 ± 0.5 °C).

Pressure, flux, temperature, and permeate concentration in filtration experiments were recorded with different feed pH.

The pressure was stable over time. Temperature variation was within 0.5 °C in each experiment. The flux had a maximum variation of 10%. The permeate concentration increased over time with the most significant increase in the filtration at pH 2.

Filtration by HY70 membrane at varying pH

Pressure, flux, temperature, and permeate concentration in filtration experiments were recorded with different feed pH.

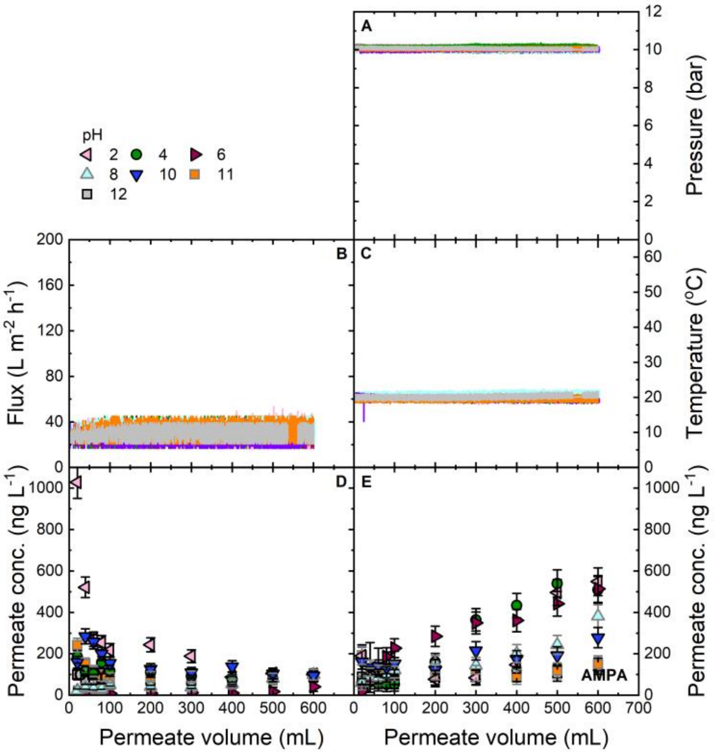

Supplementary Figure 12. Pressure, flux, feed temperature, permeate concentration by HY70 membrane (flux 30 L m^{-2} h^{-1}, initial GLY/AMPA concentration 1 µg L^{-1}, 1mM $NaHCO_3$, 10 mM NaCl, 20 ± 0.5 °C).

The pressure was stable over time. Temperature variation was within 0.5 °C in each experiment. The flux had a maximum variation of 10%. The permeate concentration increased over time.

Filtration by HY50 membrane at varying pH

Pressure, flux, temperature, and permeate concentration in filtration experiments were recorded with different feed pH.

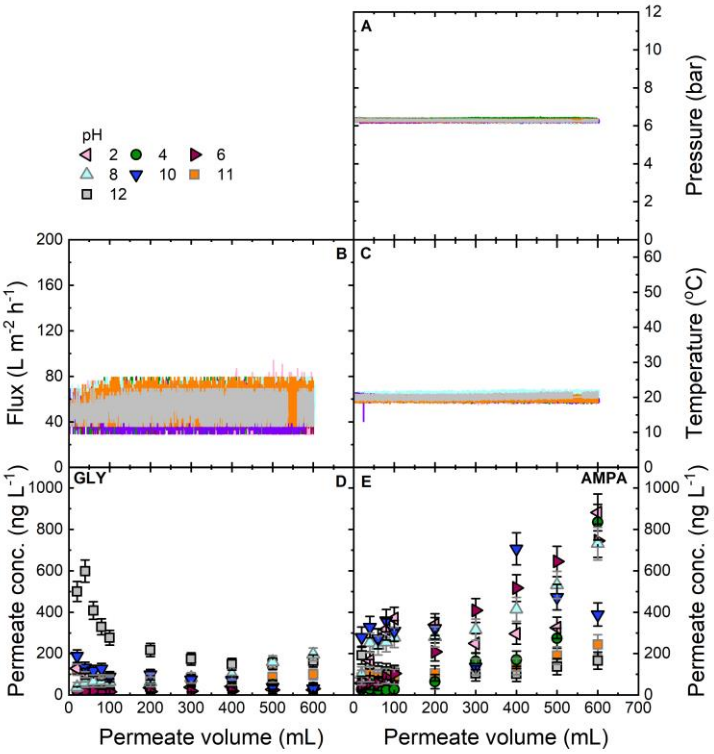

Supplementary Figure 13. Pressure, flux, feed temperature, permeate concentration by HY50 membrane (flux 50 L m^{-2} h^{-1}, initial GLY/AMPA concentration 1 µg L^{-1}, 1mM $NaHCO_3$, 10 mM NaCl, 20 ± 0.5 °C).

Filtration by HY10 membrane at varying pH

The pressure was stable over time. Temperature variation was within 0.5 °C in each experiment. The flux had a maximum variation of 15%. The permeate concentration increased over time.

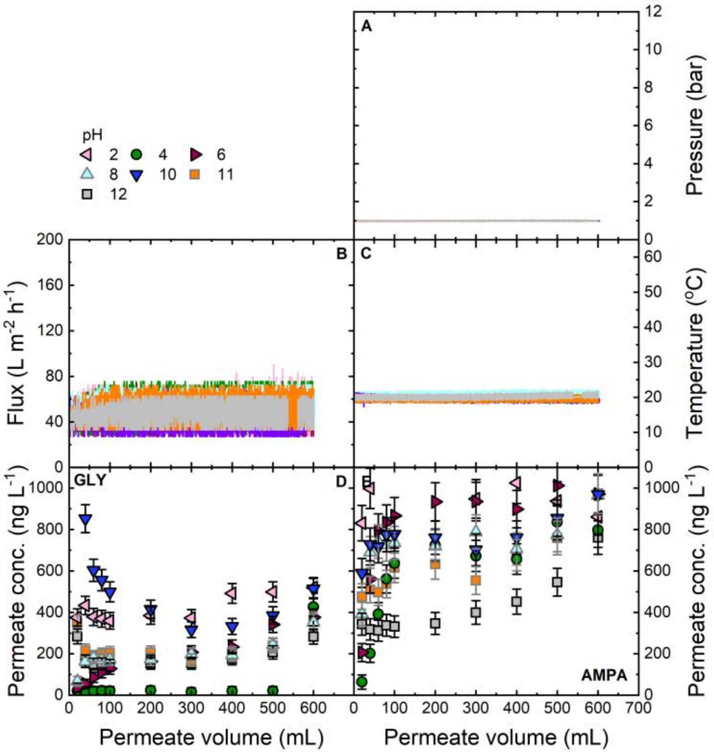

Supplementary Figure 14. Pressure, flux, feed temperature, permeate concentration by HY10 membrane (flux 50 L m^{-2} h^{-1}, initial GLY/AMPA concentration 1 µg L^{-1}, 1mM $NaHCO_3$, 10 mM NaCl, 20 ± 0.5 °C).

The pressure was stable over time. Temperature variation was within 0.5 °C in each experiment. The flux had a maximum variation of 15%. The permeate concentration increased over time.

Supplementary Method 10. Filtration protocol for NF membrane with GLY and AMPA

The filtration protocol for the NF membrane with GLY and AMPA is shown in Supplementary Table 1.

Supplementary Table 1. Filtration protocol for NF membrane

**No.**	**Step**	**Conditions (duration, volume, pressure, flow…)**
1	Membrane conditioning	Soaking the membrane coupon in NaCl 10 mM solution for 1 hour
2	Mounting the membrane in the cell	Mount the membrane coupon at the bottom of the cell with the shiny side facing the inside of the cell
3	Fixing the pressure	Open the synthetic air to flow within the cell and adjust the pressure to 9.6 bar
4	Compaction	Filtration of Milli-Q water for 1 hour at 9.6 bar
5	Pure Water Flux (before)	Filtration of Milli-Q water for 20-30 minutes at the same pressure of compaction
7	Remove water from the bottom of the cell	Use a syringe to take out the remaining water in the plastic tube of the permeate and bottom of the cell
6	Filtration of GLY/AMPA solution	Feed volume: 800 mL<br>Feed composition: 1 µg L^{-1} GLY/AMPA in a background solution (10 mM NaCl, 1 mM $NaHCO_3$)<br>Temperature: 22.1 (±0.5) °C<br>Operating conditions: 50 L m^{-2} h^{-1}, stirrer speed 400 rpm.<br>GLY/AMPA concentration in the permeate is collected.<br>Total permeate volume of 600 mL, collect 5 permeate samples of 20 mL each (until 100 mL), then 5 permeate samples of 100 mL each.
7	Remove the pressure within the cell and empty the cell	Close the on-off valve, open the pressure relief valve and let the remaining synthetic air flow out; when the pressure is zero close all the valves and dismount the cell; take the concentrate to measure conductivity and fill the cell with MilliQ water.
8	Pure Water Flux (after)	Filtration of MilliQ water for about 20-30 minutes at the same pressure used before the experiment.

Supplementary Method 11. Filtration system set-up

The dynamic filtration of UF–PBSAC was performed in a dead-end stainless steel system (Supplementary Figure 15) as described in the work of Tagliavini et al. [18].

The system consisted of a stainless steel stirred cell (designed in KIT) connected to a synthetic air tank (20% oxygen and 80% nitrogen, Alpha Gas, Air Liquid, Germany). The temperature of the membrane cell was regulated by a thermostatic circulator system composed of a chiller (LKB 2219 MultiTemp II, Bromma Germany) and a stainless-steel flexible serpentine (Water Way Engineering GmbH, Germany) wrapping around the stainless steel stirred cell. Pressure and temperature were

measured by a pressure transducer (PX219–30V85G5V, Omega Engineering, Germany) and a thermocouple (TJ2-CPSS-M60U-250-SB, Omega Engineering, Germany).

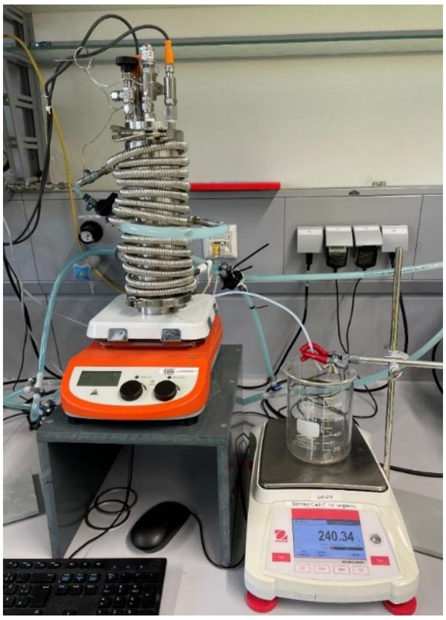

Supplementary Figure 15. UF–PBSAC filtration system with the modified bottom that allows the variable depth of PBSAC

Supplementary Method 12. Membrane characteristics

Six types of NF membranes were used in this study, including BW 30, NF 90, NF 270, HY 70, HY 50, and HY 10. Membrane properties are shown in Supplementary Table 2.

Supplementary Table 2. NF membrane types and properties (nominal MWCO, permeability, pore diameter and isoelectric point (IEP) [9, 19-21]. NF 270 and NF 90 surfaces have a net positive net charge at IEP < 4 and a negative net charge at pH > 4. HY membrane surface has a negative net charge at all pH 2−12.

**No**	**Membrane type**	**Company**	**Nominal MWCO (Da)**	**Permeability ($L\ m^{-2}\ h^{-1}\ bar^{-1}$)**	**Pore diameter (nm)**	**Zeta potential pH 8**	**Active layer materials**
1	BW 30	DuPont, USA	80−120 [a]	4 ± 1 [a]	0.41–0.51	−40 [e]	Polyamide
2	FilmTec NF 90		90−180 [b]	8 ± 2 [b]	0.44–0.63[b]	−49 [b]	Polyamide
3	FilmTec NF 270		150−340 [b]	14 ± 2 [a]	0.57–0.89 [b]	−116 [b]	Semi-aromatic piperazine-based polyamide
4	HydraCoRe 70 (HY 70)	Nitto – Hydranautics, USA	600–720 [c]	3.1 ± 0.3 [d]	1.21–1.31	−33 [d]	Sulphonated polyethersulfone
5	HydraCoRe 50 (HY 50)		1000–1500 [c]	7.8 ± 0.5 [d]	1.58–1.96	−33 [d]	Sulphonated polyethersulfone
6	HydraCoRe 10 (HY 10)		3000–3600 [c]	58 ± 8 [d]	2.83–3.12	−38 [d]	Sulphonated polyethersulfone

[a] Cai *et al.* [20], [b] Imbrogno and Schäfer [19], [c] Nominal value provided by supplier (Nitto – Hydranautics), [d] Boussouga *et al.*, [e] Idil Mouhoumed *et al.* [21], [f] not determined as IEP is below 2.

The zeta potential measurements of the membranes were not a part of this work but in previous research [9, 22, 23]. The zeta potential (ZP) of the membranes (NF 90, NF 270, HY 70, HY 50, HY 10) was calculated from the streaming potential measurements using an electrokinetic analyser

(SurPASS™ 3, Anton Paar, Austria) and following the method of Luxbacher [24]. The solution of 10 mM NaCl (VWR chemicals, purity ≥99.9 %, Germany) was used as the electrolyte at a temperature of 25 ± 2 °C. The pH of the electrolyte solution was adjusted by an integrated dosing unit using 50 mM HCl or NaOH. The membrane samples were placed between two rectangular holders (2 cm^2), facing each other with a gap height of 100 ± 2 µm. The pressure was decreasing in the range of 50–300 mbar between the inlet and the outlet of the cell, corresponding to an average flow rate of 50 mL min^{-1}. For BW 30 membrane, zeta potential was determined using an electrokinetic analyzer (EKA, Anton Paar KG, Austria) with Ag/AgCl-electrodes (SE 4.2, Senortechnik Meinsberg, Germany) in 20 mM NaCl and 1 mM $NaHCO_3$ and calculated using the Helmholtz–Smoluchowski and Fairbrother–Mastin equations [23].

Supplementary Method 13. LC-MS/MS settings and calibration of GLY and AMPA

GLY/AMPA was analyzed by LC-MS/MS (PerkinElmer, USA). The analytical method was described in detail in the previous work [25] and the instrument settings are provided in Supplementary Table 3.

Supplementary Table 3. Mass spectrometer parameters for GLY and AMPA analysis.

**Compound**	**Precursor ion (m/z)**	**Entrance voltage**	**Collision Cell Lens 2 voltage**	**Quantification ion, m/z (collision energy, eV)**	**Quantification ion, m/z (collision energy, eV)**	**Ion ratio**
GLY	168	−24	52	63 (38)	81 (22)	0.75
AMPA	110	−20	44	63 (29)	79 (33)	0.58

The calibration results of GLY and AMPA were conducted in 9 concentrations (0, 2, 5, 10, 20, 50, 200, 500, 1000 ng L^{-1}) from 50 calibration operations (Supplementary Figure 16).

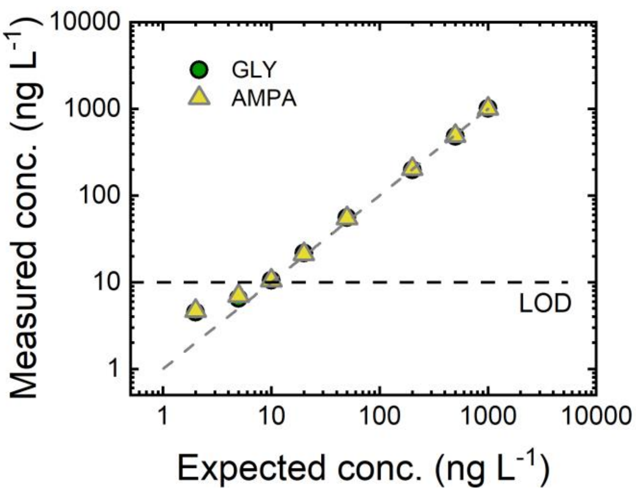

Supplementary Figure 16. Calibrations of GLY and AMPA by LC-MS/MS

The LOD was determined following a method suggested by the US Environmental Protection Agency [26] as shown below:

$$LOD = \frac{s_{blank} + 3\ \sigma_{blank}}{b} \quad \text{(S7)} \qquad LOQ = \frac{s_{blank} + 10\ \sigma_{blank}}{b} \quad \text{(S8)}$$

where s_{blank} was the measured signal of the blank, σ was the standard deviation of the blank, and b was the slope of the calibration. The LOD was calculated to be 10 ng L^{-1}.

Supplementary Method 14. Data analysis

Supplementary Table 4. Parameters in membrane adsorptive filtration of GLY / AMPA [22, 27-29].

Parameter	Formula	Eq
Water flux (J_v, L m^{-2} h^{-1})	$J_v = \frac{Q_p}{A}$ where Q_p: permeate flow rate (m^3 s^{-1}); A: active membrane area (m^2)	(S9)
Water permeability (L_p, L m^{-2} h^{-1} bar^{-1})	$L_p = \frac{J}{\Delta P}$ where ΔP: transmembrane pressure (bar)	(S10)
GLY/AMPA removal (R, %)	$R = \left(1 - \frac{c_p}{c_f}\right) \cdot 100$ where c_p and c_f: permeate and feed concentration of GLY/AMPA (ng L^{-1}), respectively.	(S11)
Specific mass adsorbed per filtration area (q_{ads}, ng cm^{-2})	$q_{ads} = \frac{V_f \cdot c_f - \sum_{i=1}^{n\ sample} V_{p,i} \cdot c_{p,i} - V_c \cdot c_c}{A}$ where V_f, V_p, V_c: volume of permeate, feed, and concentrate solutions; c_c = GLY/AMPA concentration of concentrate solutions.	(S12)
Debye length (κ^{-1}, m) [28]	$\kappa^{-1} = \sqrt{\frac{\varepsilon_0 \cdot \varepsilon_\tau \cdot R \cdot T}{F^2 \cdot \sum_i (z_i^2 \cdot C_i)}}$ where ε_0: vacuum permittivity ($8.854 \cdot 10^{-12}$ C V^{-1} m^{-1}) [30]; ε_τ: feed solution relative permittivity; R: idea gas constant (8.3143 J mol^{-1} K^{-1}); T: feed temperature (K); F: Faraday constant (96487 C mol^{-1}); z_i: valence of ion (z_{Na}^{+}=z_{Cl}^{-}=1); C_i: ion molar concentration (mol m^{-3})	(S13)
Feed solution permittivity (ε_τ) [31]	*Empirical formula* $\varepsilon_\tau = \varepsilon_W(1 - 0.17 \cdot C_{NaCl})$ where ε_W: relative permittivity of water (78.3 at 25 °C) [32]; C_{NaCl}: molar concentration of NaCl in bulk solution (mol L^{-1}).	(S14)
Debye ratio (λ) [29]	$\lambda = \frac{\kappa^{-1}}{r_p}$ where r_p: membrane pore radius (m)	(S15)
Membrane pore radius (r_p, nm) [27]	*Empirical formula* $r_p = 2.037 \cdot 10^{-11} \cdot \left(\frac{\text{nominal MWCO}}{g\ mol^{-1}}\right)^{0.53}$	(S16)
Potential at the center of NF pores (mV) [33]	$\psi(x) = \psi_d e^{-\kappa x}$ where ψ_d is the potential at the surface, κ is the Debye–Hückel parameter and κ^{-1} is the Debye length, and x is the distance from the charged surface.	(S17)

The permeate concentration, removal, and mass adsorbed are the main parameters to evaluate the performance of the NF membrane. Debye length was calculated to determine the double layer thickness. All calculations are summarized in Supplementary Table 4. Error analysis is explained in Supplementary Table 5 and Supplementary Table 6.

Supplementary Method 15. Error calculation

Error source identification

Error sources for filtration experiments are errors from feed preparation, filtration operation, and analytical errors (Supplementary Table 5).

Supplementary Table 5. Error type and sources considered in the error analysis

Type of error	Sources considered
Experimental error from filtration	Temperature variation<br>Pressure variation<br>Feed flow rate error setting
Analytical error	Error from LC-MS/MS analysis<br>Feed preparation by the operator

Feed preparation: from the use of analytical balance (± 0.1 mg, Adventurer ProAV 2102, Ohaus, Germany), volumetric flask (VWR borosilicate glass, 1000 ± 0.3 mL), measuring cylinder (VWR borosilicate glass, 250 ± 1 mL), human error (eye inspection of meniscus). Error from feed preparation: the error from pipetting and dilution is < 0.1%. Errors from the operator are minimized in the experiments. Filtration operation: pressure is adjusted by hand, so flux variation is influenced by NF permeability. The flux variation is 10% between membrane coupons. Temperature is adjusted and measured by a thermocouple (± 0.5 °C, TJ2-CPSS-M60U-250-SB, Omega Engineering, Germany). Error from filtration experiment: Temperature was controlled ± 0.3 °C, and pressure was adjusted with an error of 3%. Analytical operation: errors are calculated corresponding to > 50 measurements. The main error source is the feed solution, and the equal error source is coupon variability (resulting in flux variation) in permeate concentration. Other factors: sample degradation (if the samples are mixed with buffer and internal standard, then kept for > 1 month), ion suppression (hence conductivity is controlled), and water contamination (cannot controlled but after samples are obtained, they are capped and put in the fridge at 4 °C).

Supplementary Table 6. Error from analysis corresponding to GLY/AMPA concentration.

Concentration (c, ng L^{-1})	Error from analysis ($\Delta c_{anal.}$, ng L^{-1})
1000	60
500	45
200	30
100	20
10	12

Error from the analysis is calculated by standard deviation from calibration corresponding to > 50 measurements. The error is summarized in Supplementary Table 6. The most contributed error is from analytical error.

Error calculation and propagation

The absolute error in flux ΔJ is calculated based on the relative error in mass change with time (S18)

$$\Delta J = \sqrt{\Delta P^2 + \Delta T^2 + \Delta V^2} \quad \text{(S18)}$$

where ΔP is an error in pressure calculated by max–min variation during filtration, ΔT is an error in temperature control calculated by max–min variation during filtration, $\Delta V = \Delta m$ is an error in permeate volume (mass). The error for feed concentration includes the error of analytical errors and sample preparation errors. The absolute error in feed concentration is propagated from analytical error and feed volume error which is calculated as follows:

$$\Delta c_f = \sqrt{\Delta c_{anal.}{}^2 + \Delta c_{prep.}{}^2} \approx \Delta c_{anal.} \quad \text{(S19)}$$

where $\Delta c_{anal.}$ is the error contributed by the analysis; $\Delta c_{prep.} = c_f\left(\frac{\Delta V_f}{V_f}\right)$ is the error from feed solution preparation, which is negligible.

Therefore, with c_f = 1000 ng L^{-1}, $\Delta c_f \approx \Delta c_{anal.}$ = 60 ng L^{-1}.

The absolute error in permeate sample concentration is propagated from analytical error, solution preparation error, and filtration error (error in flux, J) which is calculated as follows:

$$\Delta c_p = \sqrt{\Delta c_{anal.}{}^2 + \Delta c_{prep.}{}^2 + \Delta c_{fil.}{}^2} = \sqrt{\Delta c_{anal.}{}^2 + c_p{}^2\left(\frac{\Delta V_p}{V_p}\right)^2 + c_p{}^2\left(\frac{\Delta J}{J}\right)^2} \quad \text{(S20)}$$

where $\Delta c_{prep.} = c_P\left(\frac{\Delta V_P}{V_P}\right)$ is the error from permeate solution preparation; $\Delta c_{fil.} = c_p\left(\frac{\Delta J}{J}\right)$ is an error contributed by the filtration process. The error from preparation (< 0.1%) is neglected.

Error from analysis ($\Delta c_{anal.}$) contributes the most, indicating that low permeate concentration c_P would have high error, with the highest error at the permeate concentration close to the LOD of the analysis. With permeate concentration, c_P = 10 ng L^{-1}, the error for permeate concentration is Δc_P = 15 ng L^{-1}. Error propagation for removal. The absolute error in removal ΔR (in %) is determined from equation (S21) with the propagation of error from feed and permeate samples.

$$\Delta R = (100 - R)\sqrt{\left(\frac{\Delta c_f}{c_f}\right)^2 + \left(\frac{\Delta c_p}{c_p}\right)^2} \quad \text{(S21)}$$

where Δc_f and Δc_p are the absolute error of the feed and permeate sample concentrations, respectively. Because ΔR is a product of $100 - R$, removal error is higher when removal is lower and vice versa.

With c_P = 900 ng L^{-1}, Δc_P = 55 ng L^{-1}, R = 10%, then ΔR = 9.8%.

With c_P = 200 ng L^{-1}, Δc_P = 32 ng L^{-1}, R = 80%, then ΔR = 3.4%.

Error propagation for specific adsorbed mass

The absolute mass adsorbed error of each feed and permeate sample (Δq_{ads}) in ng g^{-1} is calculated from the respective volume and concentration errors as calculated in equation (S22).

$$\Delta q_{ads,i} = q_i \sqrt{\left(\frac{\Delta V_i}{V_i}\right)^2 + \left(\frac{\Delta c_i}{c_i}\right)^2} \quad \text{(S22)}$$

The absolute mass adsorbed error Δq_{ads} (in ng) in adsorption is determined by the propagation of absolute mass error of feed and adsorption samples.

$$\Delta q_{ads} = \sqrt{{\Delta q_{ads,f}}^2 + \sum_{i=1}^{n} {\Delta q_{ads,i}}^2} \quad \text{(S23)}$$

With q_{ads} = 50 ng g^{-1} (corresponding R = 20%), then Δq_{ads} = 9 ng g^{-1}.

Supplementary References

[1] B. Van der Bruggen, J. Schaep, D. Wilms, C. Vandecasteele, Influence of molecular size, polarity and charge on the retention of organic molecules by nanofiltration, Journal of Membrane Science, 156 (1999) 29-41.

[2] G. Hurwitz, G.R. Guillen, E.M.V. Hoek, Probing polyamide membrane surface charge, zeta potential, wettability, and hydrophilicity with contact angle measurements, Journal of Membrane Science, 349 (2010) 349-357.

[3] L.D. Nghiem, A.I. Schäfer, M. Elimelech, Role of electrostatic interactions in the retention of pharmaceutically active contaminants by a loose nanofiltration membrane, Journal of Membrane Science, 286 (2006) 52-59.

[4] W.R. Bowen, J.S. Welfoot, Modelling the performance of membrane nanofiltration—critical assessment and model development, Chemical Engineering Science, 57 (2002) 1121-1137.

[5] S. You, J. Lu, C.Y. Tang, X. Wang, Rejection of heavy metals in acidic wastewater by a novel thin-film inorganic forward osmosis membrane, Chemical Engineering Journal, 320 (2017) 532-538.

[6] H.A. Pereira, P.R.T. Hernandes, M.S. Netto, G.D. Reske, V. Vieceli, L.F.S. Oliveira, G.L. Dotto, Adsorbents for glyphosate removal in contaminated waters: a review, Environmental Chemistry Letters, 19 (2021) 1525-1543.

[7] A.L. Valle, F.C.C. Mello, R.P. Alves-Balvedi, L.P. Rodrigues, L.R. Goulart, Glyphosate detection: methods, needs and challenges, Environmental Chemistry Letters, 17 (2019) 291-317.

[8] S. Paul, W.F. Meggitt, P. Donald, Adsorption, mobility, and microbial degradation of glyphosate in the soil, Weed Science, 23 (1975) 229-234.

[9] Y.-A. Boussouga, T. Okkali, T. Luxbacher, A.I. Schäfer, Chromium (III) and chromium (VI) removal and organic matter interaction with nanofiltration, Science of The Total Environment, 885 (2023) 163695.

[10] S.S. Sablani, M.F.A. Goosen, R. Al-Belushi, M. Wilf, Concentration polarization in ultrafiltration and reverse osmosis: a critical review, Desalination, 141 (2001) 269-289.
[11] G.B. van den Berg, I.G. Rácz, C.A. Smolders, Mass transfer coefficients in cross-flow ultrafiltration, Journal of Membrane Science, 47 (1989) 25-51.
[12] A. Schäfer, A.G. Fane, T.D. Waite, Nanofiltration: Principles and Applications, Elsevier, 2005.
[13] M.C. Porter, Concentration polarization with membrane ultrafiltration, Product R&D, 11 (1972) 234-248.
[14] C.P. Koutsou, A.J. Karabelas, Shear stresses and mass transfer at the base of a stirred filtration cell and corresponding conditions in narrow channels with spacers, Journal of Membrane Science, 399-400 (2012) 60-72.
[15] C.K. Colton, K.A. Smith, Mass transfer to a rotating fluid. Part II. Transport from the base of an agitated cylindrical tank, AIChE Journal, 18 (1972) 958-967.
[16] M.W. Feyereisen, D. Feller, D.A. Dixon, Hydrogen bond energy of the water dimer, The Journal of Physical Chemistry, 100 (1996) 2993-2997.
[17] R. Epsztein, R.M. DuChanois, C.L. Ritt, A. Noy, M. Elimelech, Towards single-species selectivity of membranes with subnanometre pores, Nature Nanotechnology, 15 (2020) 426-436.
[18] M. Tagliavini, P.G. Weidler, C. Njel, J. Pohl, D. Richter, B. Böhringer, A.I. Schäfer, Polymer-based spherical activated carbon – ultrafiltration (UF-PBSAC) for the adsorption of steroid hormones from water: Material characteristics and process configuration, Water Research, 185 (2020) 116249.
[19] A. Imbrogno, A.I. Schäfer, Comparative study of nanofiltration membrane characterization devices of different dimension and configuration (cross flow and dead end), Journal of Membrane Science, 585 (2019) 67-80.
[20] Y.-H. Cai, A.I. Schäfer, Renewable energy powered membrane technology: Impact of solar irradiance fluctuation on direct osmotic backwash, Journal of Membrane Science, 598 (2020) 117666.
[21] E. Idil Mouhoumed, A. Szymczyk, A. Schäfer, L. Paugam, Y.H. La, Physico-chemical characterization of polyamide NF/RO membranes: Insight from streaming current measurements, Journal of Membrane Science, 461 (2014) 130-138.
[22] Y.-A. Boussouga, H. Than, A.I. Schäfer, Selenium species removal by nanofiltration: Determination of retention mechanisms, Science of The Total Environment, 829 (2022) 154287.
[23] L.A. Richards, M. Vuachère, A.I. Schäfer, Impact of pH on the removal of fluoride, nitrate and boron by nanofiltration/reverse osmosis, Desalination, 261 (2010) 331-337.
[24] T. Luxbacher, The ZETA guide: Principles of the streaming potential technique, Anton Paar GmbH: Graz, Austria, (2014).
[25] P.B. Trinh, A.I. Schäfer, Adsorption of glyphosate and metabolite aminomethylphosphonic acid (AMPA) from water by polymer-based spherical activated carbon (PBSAC), Journal of Hazardous Materials, (2023) 131211.
[26] US Environmental Protection Agency, 2016, Definition and procedure for the determination of the method detection limit, revision 2, accessed 08.08 2023, https://www.epa.gov/sites/default/files/2016-12/documents/mdl-procedure_rev2_12-13-2016.pdf.
[27] E. Worch, Eine neue Gleichung zur Berechnung von Diffusionskoeffizienten gelöster Stoffe, Vom Wasser, 81 (1993) 289–297.
[28] P. Atkins, J. DePaula, J. Keeler, Atkins' Physical Chemistry, Oxford University Press, Oxford, 2006.
[29] J.K. Bungay, Synthetic membranes: Science, engineering and applications, Springer Science & Business Media, 2012.
[30] R. Shang, A.R.D. Verliefde, J. Hu, Z. Zeng, J. Lu, A.J.B. Kemperman, H. Deng, K. Nijmeijer, S.G.J. Heijman, L.C. Rietveld, Tight ceramic UF membrane as RO pre-treatment: The role of electrostatic interactions on phosphate rejection, Water Research, 48 (2014) 498-507.
[31] K. Nörtemann, J. Hilland, U. Kaatze, Dielectric properties of aqueous NaCl solutions at microwave frequencies, The Journal of Physical Chemistry A, 101 (1997) 6864-6869.
[32] D.G. Archer, P.-m. Wang, The dielectric constant of water and Debye‐Hückel limiting law slopes, Journal of Physical and Chemical Reference Data, 19 (1990) 371-411.
[33] S. Bhattacharjee, DLS and zeta potential – What they are and what they are not?, Journal of Controlled Release, 235 (2016) 337-351.
